# Post-transplant diabetes is associated with late systolic left ventricular dysfunction after heart transplantation

**DOI:** 10.1016/j.jhlto.2026.100622

**Published:** 2026-06-30

**Authors:** Anna Werther Evaldsson, Ashkan Labaf, Grunde Gjesdal, Raluca Jumatate, Oscar Östberg Braun, J. Gustav Smith, Annika Ingvarsson

**Affiliations:** a Department of Clinical Sciences, Lund, Cardiology, and the Section for Heart Failure and Valvular Disease, Skåne University Hospital, Lund University, Lund, Sweden; b Department of Molecular and Clinical Medicine, Institute of Medicine, University of Gothenburg, Gothenburg, Sweden

**Keywords:** heart transplantation, post-transplant diabetes mellitus, global longitudinal strain, left ventricular function

## Abstract

**Background:**

Post-transplant diabetes mellitus (PTDM) is common after orthotopic heart transplantation (OHT), but its long-term impact on myocardial function is unclear. We aimed to evaluate the effects of PTDM on left ventricular (LV) systolic function.

**Methods:**

We prospectively studied 102 OHT recipients with echocardiographic follow-up at 1-, 3-, and 5-year post-transplant. Patients were classified according to PTDM status at 1 year. LV systolic function was assessed using ejection fraction (EF) and global longitudinal strain (GLS). Linear mixed-effects models were used to evaluate longitudinal associations, adjusting for age, sex, systolic blood pressure, and cardiac allograft vasculopathy (CAV).

**Results:**

PTDM was present in 36% of patients. At 1-year, EF and GLS were similar between groups. At 3 years, patients with PTDM exhibited significantly lower systolic function as assessed by GLS (−14.9±2.8 vs. −16.0±3.0%, *p*=0.022), and at 5 years, EF was also significantly lower (52±4 vs. 57±7%, *p*=0.002). In longitudinal analyses, PTDM was associated with persistently impaired GLS without a significant interaction with time (*p*=0.192). In contrast, EF demonstrated a significant PTDM×time interaction (*p*=0.039), reflecting a greater decline over time in patients with PTDM. These associations remained significant after adjustment for CAV, which was not independently associated with GLS or EF.

**Conclusion:**

PTDM after OHT is associated with early and persistent impairment in myocardial deformation, followed by a progressive decline in systolic function. These findings suggest a metabolically mediated myocardial effect independent of chronic graft failure and highlight the importance of early detection and management of PTDM in heart transplant recipients.

## Graphical abstract

**Figure:**
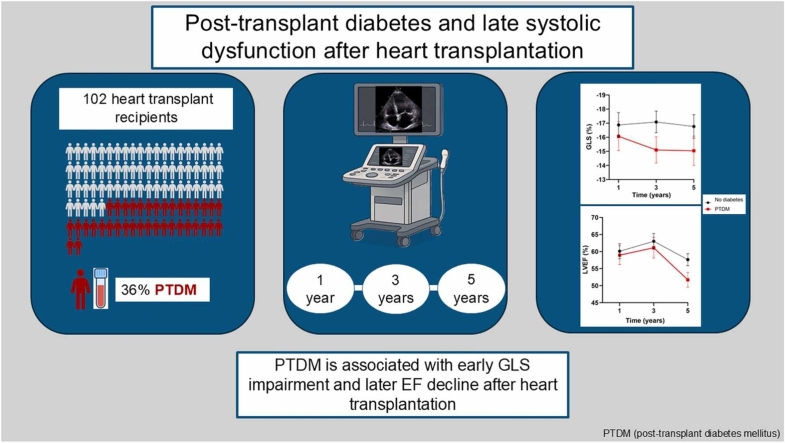

In selected cases, orthotopic heart transplantation (OHT) is an established treatment option for patients with end-stage advanced heart failure. Lifelong immunosuppressive therapy is required to prevent graft rejection. However, commonly used immunosuppressive agents, such as tacrolimus, are associated with an increased risk of post-transplant diabetes mellitus (PTDM).^1^, ^2^, ^3^ Corticosteroids, among the earliest immunosuppressive agents used in transplantation, remain an important component of modern immunosuppressive regimens due to their potent anti‑inflammatory and immunosuppressive effects.^1^ Consequently, efforts have focused on balancing rejection risk against adverse metabolic effects.

In non-transplant populations, numerous studies have described echocardiographic features of diabetic cardiomyopathy, including left ventricular (LV) hypertrophy, diastolic dysfunction, and impaired systolic function.^4^, ^5^ In contrast, the impact of diabetes on cardiac structure and function after OHT remains to be fully elucidated. The increased use of steroid-free immunosuppressive regimens might impact the prevalence of PTDM, but the presence of PTDM remains a challenge in OHT patients.^3^ Given the vulnerability of this patient population, identifying early markers of subclinical ventricular dysfunction associated with PTDM is particularly important.

A better understanding of the effects of PTDM on cardiac allograft structure and function may improve early risk stratification and inform clinical management in transplant recipients. Therefore, this study aimed to evaluate the longitudinal effects of PTDM on LV function and remodeling using serial echocardiography during the first 5 years after OHT.

## Material and methods

### Study design

Echocardiographic, hemodynamic, angiographic, and medication data from 140 OHT recipients followed at our center between 2004 and 2022 were collected. All examinations were performed as part of routine clinical follow-up protocol at 1-, 3-, and 5-year post-transplantation. At the 1-year follow‑up, transthoracic echocardiography (TTE), right heart catheterization (RHC), and coronary angiography were conducted, whereas echocardiography and angiography were performed at the 3- and 5-year visits. A comprehensive TTE protocol previously described,^6^ was used to assess differences in left-heart structure and function, with dedicated evaluations at 3- and 5-year post-OHT.

Medication data were obtained from medical records at the 1-year follow‑up. At all 3 follow-up time points (1-, 3-, and 5-years), medical records and angiography were reviewed to determine the presence of cardiac allograft vasculopathy (CAV).

Patients were categorized according to the presence or absence of PTDM at 1 year after transplantation. PTDM status was extracted from medical records according to standard clinical practice, including HbA1c ≥6.5% (48 mmol/mol),^7^ and was treated as a fixed exposure in all analyses.

**Figure 1:**
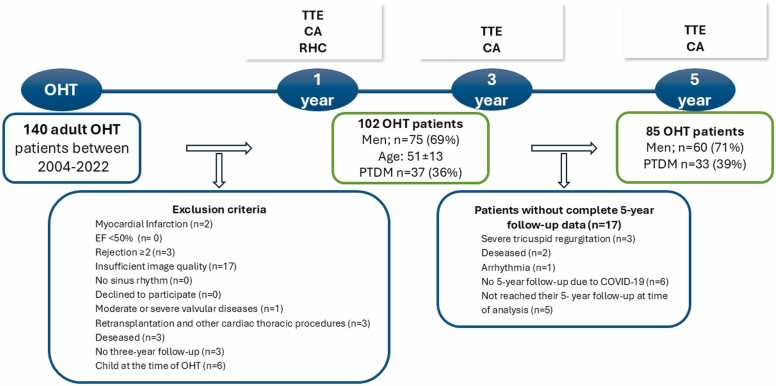
Study flow chart of the OHT cohort. Adult patients undergoing OHT between 2004 and 2022 were screened and followed longitudinally with standardized post‑transplant evaluations at 1-, 3-, and 5-years. RHC, TTE, CA, EF, PTDM. CA, coronary angiography; EF, left ventricular ejection fraction; OHT, orthotopic heart transplantation; PTDM, post-transplant diabetes mellitus; RHC, right heart catheterization; TTE, transthoracic echocardiography

The study cohort was initially defined as patients with complete echocardiographic data at both 1-year and 3-year. After applying the exclusion criteria, Figure 1, 102 patients were eligible. Five-year data were available for 85 patients, with reasons for missing follow-up shown in Figure 1.

### Echocardiography

Conventional 2D echocardiography was performed using Philips iE33, Philips Epic CVx (Philips Healthcare, Eindhoven, Netherlands), or Vivid E95 systems (GE Healthcare, Horten, Norway). Standard echocardiographic parameters were measured according to American Society of Echocardiography (ASE) guidelines.^8^ LV systolic function was assessed using ejection fraction (EF) and global longitudinal strain (GLS). GLS is reported as a negative value, with more negative values indicating poorer deformation. Measurements were indexed to body surface area when appropriate. Diastolic function parameters were included according to contemporary ASE recommendations.^9^

All data were analyzed using IntelliSpace Cardiovascular 5 (version 5.2; Philips Medical Systems Nederland B.V., Best, The Netherlands). To minimize inter-vendor variability, all strain analyses were performed by a single experienced senior sonographer (A.I.) using vendor-independent software (Ultrasound Workspace/TomTec Imaging Systems, version 52.00; TomTec, Unterschleissheim, Germany). Ejection fraction measurements were performed by 2 experienced observers (A.I. and A.W.E.), each with 19 years of experience.

### Right heart catheterization

RHC was performed in the supine position using a Swan–Ganz catheter. Pulmonary artery pressures, mean right atrial pressure, and pulmonary arterial wedge pressure (PAWP) were recorded. PAWP was measured during free breathing over multiple cardiac cycles. Cardiac output and stroke volume were determined using thermodilution. Systemic blood pressure was measured non-invasively. Elevated left-sided filling pressure was defined as PAWP > 15 mmHg.^10^

### Coronary angiography

Coronary angiography was performed according to routine clinical practice. CAV was assessed dichotomously as present or absent, without grading of severity.

### Statistics

Data are presented as mean ± standard deviation (SD) or median with interquartile range [IQR], depending on distribution. Normality was assessed visually using histograms. Patients were categorized according to the presence or absence of PTDM 1-year after transplantation.

Between-group comparisons at individual follow-up time points were performed using Student’s t-test or the Mann–Whitney U test, as appropriate. Categorical variables were compared using chi-square test or Fisher’s exact test when expected cell counts were <5.

To evaluate longitudinal associations between PTDM at 1-year and repeated measures of LV systolic function across 1-, 3-, and 5-year intervals, linear mixed-effects models were used. These models enabled the inclusion of all available observations across the follow-up period. A random intercept for each subject was included to account for within-patient correlation. Models included PTDM group, time, and the PTDM × time interaction as fixed effects and were adjusted for age, sex, and systolic blood pressure; secondary models included HbA1c and CAV. Estimated marginal means derived from the mixed-effects models were used for graphical illustration of longitudinal trajectories. A 2-tailed *p*-value <0.05 was considered statistically significant. All analyses were performed using IBM SPSS Statistics, version 25 (IBM Corp., Armonk, NY, USA).

### Ethics

The study received approval from the regional Swedish Ethical Review Authority (Dnr 2022–02631–01, 2024–01957–02) and adheres to the principles of the Declaration of Helsinki and Istanbul, as well as the ISHLT statement on transplant ethics. Patients provided written informed consent.

## Results

### Patient characteristics

Patient characteristics and medical treatment at 1-year follow‑up are summarized in Table 1. The cohort consisted of 74% men, mean age 51 ± 13 years. PTDM was present in 37 patients (36%). PTDM status was defined at the 1-year follow-up and used as a fixed classification for all subsequent analyses. Although some patients changed glycemic status during follow-up (5 no longer fulfilled diagnostic criteria at later follow-up visits, and 7 developed diabetes by the 3-year follow-up) analyses were performed according to 1-year status.

**Table 1:** Baseline and Hemodynamic Characteristics at 1-Year Follow-up

	Patient cohort (n=102)	No PTDM (n=65)	PTDM (n=37)	*p*
*Demographic and clinical characteristics*				
Sex (men)	75 (74)	53 (82)	22 (59)	**0.015**
Age (years)	51±13	50±13	53±13	0.620
BSA (m^2^)	2.0±0.2	2.0±0.2	1.9±0.2	0.633
HbA1c (mmol/l)	39±7	37±6	42±9	**<0.001**
CAV	23 (23)	13 (20)	10 (27)	0.517
HR (beats/min)	85±10	86±11	84±10 (n.s)	0.532
SBP (mmHg)	135±14	131±14	138±14	**0.028**
DBP (mmHg)	82±10	82±10	83±10	0.109
*Hemodynamics (RHC)*				
SPAP (mmHg)	24±7	24±7	24±7	0.797
mPAP (mmHg)	15±4	15±4	16±4	0.360
PAWP (mmHg)	7±4	6±4	7±4	0.444
mRAP (mmHg)	2 [1-4]	2 [1-4]	3 [0-4]	0.839
CO (L/min)	5.8±1.5	6.2±1.6	5.4±0.6	**<0.004**
SV (ml)	73±14	76±15	69±13	**0.035**
PVR (WU)	1.4±1.3	1.4±0.6	1.5±0.7	0.721
*Medication*				
Prednisolone	95 (94)	60 (92)	35 (95)	1.000
Tacrolimus	70 (69)	40 (62)	30 (81)	**0.041**
Mycophenolate mofetil	86 (85)	55 (85)	31 (84)	0.769
Everolimus	3 (3)	2 (3)	1 (3)	1.000
Cyclosporine	39 (39)	28 (43)	11 (30)	0.163
Azathioprine	10 (10)	8 (13)	2 (5)	0.250
Diltiazem	54 (32)	34 (53)	20 (54)	0.928
Beta blockers	46 (46)	21 (33)	25 (68)	**<0.001**
Statin	85 (84)	55 (86)	30 (81)	0.520
ASA	63 (62)	40 (62)	22 (60)	0.762
ACE inhibitors / ARBs	30 (30)	21 (32)	9 (24)	0.368
Loop diuretics	35 (35)	19 (30)	16 (43)	0.168
Thiazide diuretics	3 (3)	2 (3)	1 (3)	1.000
Potassium-sparing diuretics	5 (5)	4 (6)	1 (3)	0.650
Insulin	23 (23)	0 (0)	23 (62)	**<0.001**
Metformin	1 (2)	0	1 (3)	0.366
DPP-4 inhibitors	1 (1)	0	1 (3)	0.366
Felodipin/amlodiopin	8 (8)	2 (3)	6 (16)	**0.019**

Values are represented as mean±SD, median [IQR] or number (per cent). ACE, angiotensin converting enzyme; ARB, angiotensin receptor blockers; ASA, Acetylsalicylic acid; CAV, Cardiac allograft vasculopathy; CO, cardiac output; DBP, diastolic blood pressure; DPP, Dipeptidyl peptidase; HR, heart rate; mPAP, mean pulmonary arterial pressure; mRAP, mean right atrial pressure; PAWP, pulmonary arterial wedge pressure; PTDM, post-transplant diabetes mellitus; PVR, pulmonary vascular resistance; RHC, right heart catheterization; SBP, systolic blood pressure; SPAP, systolic pulmonary arterial pressure; SV, stroke volume

Mean HbA1c in the PTDM group at 1- year was 42 mmol/mol, reflecting active glucose-lowering therapy. Medication use differed between groups, with more frequent use of tacrolimus, β‑blockers, insulin, and calcium-channel blockers in PTDM patients. No patients were treated with sodium–glucose cotransporter‑2 (SGLT‑2) inhibitors.

At the 1-year follow-up, patients with PTDM had higher systolic blood pressure compared with those without PTDM (137±14 vs. 131±14 mmHg, *p*=0.028). These differences were not observed at the 3- and 5-year follow-up. Consistent with the clinical diagnosis, HbA1c levels were significantly higher in the PTDM group at all follow-up visits (1-year, p < 0.001; 3-year, *p* < 0.001; and 5-year, *p* = 0.01).

### Hemodynamics and CAV

Right heart catheterization data at 1-year are shown in Table 1. The interval between RHC and echocardiography was 1 day [1,2]. Pulmonary pressures, LV filling pressures, and PVR were within normal ranges in both groups. There were no differences in CAV between the groups, as shown in Table 2. PTDM at 1 year was not associated with CAV at either 3 years (*p* = 0.093) or 5 years (*p* = 0.256).

**Table 2:** Echocardiographic Characteristics Illustrating Left Ventricular Function and Diastolic Parameters at 1-, 3-, and 5-Year Follow-up

	1-year follow-up (n=102)			3-year follow-up (n=102)			5-year follow-up (n=85)		
	**No PTDM**	**PTDM**	***p*-value**	**No PTDM**	**PTDM**	***p*-value**	**No PTDM**	**PTDM**	***p*-value**
CAV	13 (23)	10 (27)	0.522	21 (33)	17 (43)	0.095	18 (34)	14 (44)	0.262
HR (beats/min)	86±11	85±10	0.532	82±9	85±11	0.070	83±11	83±9	0.862
SBP (mmHg)	131±14	137±14*	**0.028**	134±17	133±19	0.760	134±18	132±17	0.726
DBP (mmHg)	81±10	84±11	0.109	83±10	84±9	0.395	83±11	83±14	0.817
HbA1c (mmol/l)	37±5	43±8***	**<0.001**	37±5	44±13***	**<0.001**	38±6	43±9*	**0.010**
Left ventricular parameters									
IVSd (mm)	10.4±2.4	10.8±1.8	0.369	10.7±2.3	10.8±1.6	0.938	10.7±2.1	10.4±1.8	0.579
LVIDd (mm)	45±6	44±9	0.292	43±11	44±7	0.471	45±6	44±7	0.556
LVIDs (mm)	29±5	29±6	0.974	30±8	29±6	0.193	28±6	28±4	0.975
LVPWd (mm)	10.1±1.9	10.2±1.8	0.811	10.4±1.9	10.6±1.8	0.593	10.4±2.8	10.1±1.6	0.601
LV mass (g)	165±50	161±40	0.681	167±58	167±49(36)	0.827	164±38	163±48	0.964
LVEDV (ml)	96±21	96±22	0.907	100±18	93±19	0.079	94±26	93±25	0.776
EF (%)	59±7	59±9	0.829	62±10	61±8	0.533	57±7	52±4**	**0.002**
GLS (%)	-16.1±3.0	-16.0±3.1	0.843	-16.4±3.0	-14.9±2.8*	**0.022**	-16.2±3.0	-15.2±2.2	0.146
GLS, men (%)	-15.6±3.1 (n=40)	**-**15.1±3.0 (n=18)	0.568	-15.8±2.7 (n=44)	-13.7±2.8** (n=17)	**0.009**	**-**15.6±2.9 (n=35)	**-**14.9±2.1 (n=13)	0.409
GLS, women (%)	-18.2±2.1 (n=10)	-17.1±3.0 (n=14)	0.318	-18.6±2.5 (n=11)	-16.3±2.1* (n=14)	**0.026**	**-**18.3±2.5 (n=10)	**-15.5**±2.3* (n=13)	**0.012**
SV (ml)	60±13	54±10*	**0.042**	61±15	57±12	0.275	59±12	57±14	0.488
CO (ml/min)	5.1±1.1	4.5±0.9**	**0.008**	4.9±1.1	4.8±1.1	0.867	4.9±1.2	4.7±1.3	0.572
Diastolic parameters									
LAVi (ml/m^2^)	36.5±10.7	38.4±11.0	0.408	35.7±10.0	35.7±11.0	0.983	39±12	38±12	0.811
MVE (cm/s)	79.5±23.3	78.2±17.4	0.774	80.6±19.0	76.0±18.6	0.774	83.6±23.5	77.2±16.8	0.185
MVA (cm/s)	47.6±10.2	43.4±9.6	0.075	43.0±10.5	42.7±12.1	0.890	43.7±11.0	42.0±10.6	0.544
E/A	1.7±0.5	1.8±0.4	0.426	1.9±0.5	1.8±0.5	0.387	2.0±0.7	2.0±0.9	0.912
Lateral é (cm/s)	12.5±3.3	12.0±2.4	0.526	12.6±2.8	11.7±2.7	0.172	11.6±2.4	11.4±2.6	0.720
Septal é (cm/s)	7.8±2.4	7.3±2.2	0.355	7.6±1.9	7.8±2.4	0.767	7.5±1.7	7.0±2.2	0.235
E/é	8.4±2.8	8.4±2.8	0.915	8.6±3.0	8.2±2.7	0.445	9.4±5.0	9.9±5.8	0.681
TR max gradient (mmHg)	23.3±5.2	23.3±4.0	1.000	22.6±6.8	22.5±4.8	0.911	22.6±5.1	20.9±3.7	0.177

Values are presented as mean ± SD. Statistical comparisons between PTDM and no-PTDM groups were performed using one-way ANOVA. Significance levels are denoted as *p < 0.05, **p < 0.01, and ***p < 0.001.

CO, Cardiac output; DBP, diastolic blood pressure; EF, ejection fraction; GLS, global longitudinal strain; HBA1c, hemoglobin A1c glycated hemoglobin; HR, heart rate; IVSd, intraventricular septal diameter; LAVI, left atrial volume index; LVEDVi, LV end-diastolic volume index; LV, left ventricle; LVIDd, LV internal diameter in diastole; LVIDs, LV internal diameter in systole; LVPWd, LV posterior wall diameter; MVE, mitral valve E velocity; MVA, mitral valve A velocity; PTDM, post-transplant diabetes mellitus; SBP, systolic blood pressure; SV, Stroke volume; TRmaxv, Tricuspid regurgitation maximum velocity.

### Echocardiographic findings

**Figure 2:**
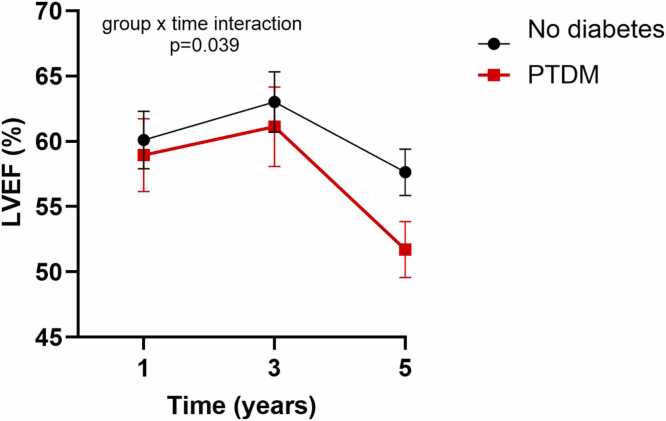
Longitudinal GLS according to PTDM status at 1 year after heart transplantation. Values represent estimated marginal means derived from linear mixed‑effects models adjusted for age, sex, and systolic blood pressure. PTDM was associated with lower GLS throughout follow‑up (*p* = 0.003). GLS, global longitudinal strain; PTDM, post‑transplant diabetes mellitus

**Figure 3:**
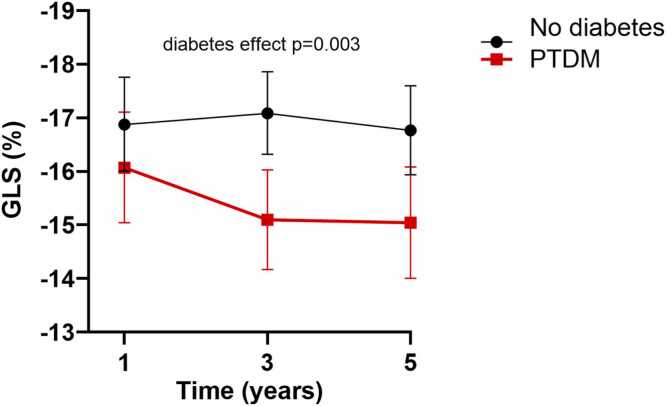
Longitudinal left ventricular EF according to PTDM status at 1 year after transplantation. Values represent estimated marginal means derived from linear mixed‑effects models adjusted for age, sex, and systolic blood pressure. A significant PTDM × time interaction was observed (*p* = 0.039), reflecting a greater decline in LVEF over time among patients with PTDM compared with those without PTDM. EF, ejection fraction; LVEF, left ventricular ejection fraction; PTDM, post‑transplant diabetes mellitus

Echocardiographic parameters of LV structure and function are presented in Table 2. Cardiac structural parameters, including LV diameters, wall thickness, LV mass, and chamber volumes, did not differ between groups at any time point (*p* > 0.05) and were within normal ranges.^6^, ^8^ GLS was lower at the 3-year follow-up in the PTDM group (−14.9±2.8 vs. −16.0±3.0 %, *p*=0.022) and at 5 years EF was also significantly lower in these patients (52±4 vs. 57±7 %, *p*=0.002), Figure 2, Figure 3.

Across all time points, no statistically significant differences were observed between groups for left atrial volume index or any diastolic function parameters. All patients had normal left atrial volumes compared with reference values for OHT patients.^6^ They also showed normal E/A, é velocities, E/é, and pulmonary pressures.

In sex‑stratified analyses by recipient gender, women had better GLS than men at all time points (all *p* ≤ 0.049). In men, GLS was similar between the PTDM and non‑PTDM groups at 1- and 5-years (−15.1±3.0 vs. −15.6±3.1 %, *p*=0.568 and −14.9±2.1 vs −15.6±2.9 %, *p*=0.409, respectively), but lower in the PTDM group at 3-years (−13.7±2.8 vs −15.8±2.7 %, *p* = 0.009). In women, no difference was detected at 1-year (PTDM −17.1±3.0%, vs. non-PTDM −18.2±2.1%, *p*=0.318); however, the PTDM group had lower GLS at both 3- and 5- years (−16.3±2.1% vs. −18.6±2.5%, *p*=0.026 and −15.5±2.3% vs. −18.3±2.5%, *p* = 0.012, respectively).

### Longitudinal analyses

In linear mixed-effects models adjusted for age, sex, and systolic blood pressure, PTDM at 1-year was associated with impaired LV systolic function during follow-up, as summarized in Table 3.

**Table 3:** Linear Mixed-Effects Models Adjusted for Age, Sex, and Systolic Blood Pressure

Outcome		β	95% CI	*p*-value
GLS				
	PTDM at 1 year	0.81	−0.53 to 2.15	0.234
	Time	-	-	0.340
	PTDM x time	-	-	0.192
	Male sex	2.30	1.24-3.37	<0.001
	Systolic BP	-0.005	0.025-0.016	0.665
EF				
	PTDM at 1 year	-1.15	−4.56 to 2.26	0.507
	Time	-	-	<0.001
	PTDM (overall)	-	-	0.015
	PTDM x time	-	-	0.039
	Time x PTDM (at 3 years)	-0.82	−4.79 to 3.14	0.683
	Time x PTDM (at 5 years)	-4.89	−8.79 to 1.00	0.014
	Male sex	-2.68	−5.32 to 0.04	0.047
	Systolic BP	-0.004	−0.061 to 0.053	0.890

*Time was treated as a categorical variable (1-, 3-, and 5-year follow‑up), with 1 year as the reference category. GLS values are presented as absolute values; less negative values indicate worse systolic function. BP, blood pressure; EF, ejection fraction; GLS, global longitudinal strain; PTDM, post-transplant diabetes mellitus

For GLS, there was no significant difference between groups at the 1-year time point (β = 0.81, 95% CI −0.53 to 2.15, *p* = 0.234). However, PTDM was associated with less negative GLS values across follow-up (overall effect *p* = 0.003), without evidence of a differential temporal trajectory between groups (PTDM × time, *p* = 0.192), indicating a relatively stable between-group difference over time, Figure 2.

For EF, there was also no significant difference between groups at 1-year (β = −1.15, 95% CI −4.56 to 2.26, *p* = 0.507). However, both time (*p* < 0.001) and PTDM (*p* = 0.015) were associated with EF during follow-up. Importantly, a significant interaction between PTDM and time was observed (*p* = 0.039), indicating a differential longitudinal trajectory. This was reflected by a greater decline in EF among patients with PTDM, with a significant between-group difference emerging at 5-year follow-up (β = −4.89, 95% CI −8.79 to −1.00, *p* = 0.014), Figure 3.

In secondary models including CAV, the association between PTDM and GLS remained significant (*p* = 0.006), while CAV itself was not associated with GLS (*p* = 0.931). For EF, the PTDM × time interaction remained significant after adjustment for CAV (*p* = 0.029), and CAV was not independently associated with EF (*p* = 0.337).

## Discussion

The principal finding of this study is a temporal dissociation between 2 markers of left ventricular systolic function in heart transplant recipients with PTDM. GLS was already impaired at 3 years post-transplantation and remained stable thereafter, whereas EF declined progressively and became significant at 5 years, despite preserved LV size and wall thickness. Although EF is known to be subject to intra- and inter-observer variability, measurements were performed by 2 experienced observers. This pattern, observed independently of CAV, suggests that subclinical myocardial deformation precedes overt systolic dysfunction.

The prevalence of PTDM in OHT patients has been reported to range from 21% to 35%.^11^, ^12^ Variability in these estimates is mainly attributable to differences in diagnostic criteria, screening strategies, and immunosuppressive regimens. Previous studies indicate that approximately 21% of recipients develop PTDM within 5 years post-OHT and that PTDM is associated with adverse outcomes, including renal dysfunction, mortality, and re-transplantation.^11^ In our cohort, 36% developed PTDM, which is slightly higher than previously reported and may reflect differences in recipient characteristics or contemporary immunosuppressive strategies.

CAV is a major determinant of long-term graft dysfunction after OHT. However, in our study, CAV prevalence did not differ between patients with and without PTDM, and CAV was not independently associated with GLS or EF in longitudinal analyses. Furthermore, adjustment for CAV did not attenuate the association between PTDM and myocardial dysfunction suggesting the impairment is unlikely driven by macrovascular disease. Although cardiac magnetic resonance imaging could provide additional characterization of myocardial tissue and function, repeated echocardiographic assessments allowed for longitudinal evaluation in this cohort. Instead, these findings support the involvement of metabolic or microvascular mechanisms affecting the myocardium.

Our findings are consistent with the concept of diabetic cardiomyopathy in non-transplant populations, in which myocardial dysfunction often precedes structural remodeling. GLS is a sensitive marker of early systolic dysfunction and has been shown to detect subclinical myocardial impairment before reductions in EF occur.^13^ In our study, persistent GLS impairment among PTDM patients, despite preserved EF at earlier follow-up, supports the role of strain imaging as an early indicator of allograft dysfunction.

Several mechanisms may contribute to these findings, including insulin resistance, myocardial lipid accumulation, microvascular dysfunction, and mitochondrial impairment, all of which have been implicated in diabetic cardiomyopathy.^14^ In transplanted hearts, these processes may be further exacerbated by systemic inflammation and metabolic stress related to immunosuppressive therapy, potentially leading to early alterations in myocardial deformation before overt systolic dysfunction develops.

Importantly, longitudinal analyses demonstrated that PTDM was associated with persistently impaired GLS without a significant PTDM × time interaction, indicating a stable between‑group difference. In contrast, EF showed a significant interaction with time, with a greater decline among patients with PTDM. This pattern suggests a sequence in which metabolic disturbances initially impair myocardial deformation, followed by later reductions in global systolic function. Notably, although HbA1c differed between groups, absolute values were modest, and no meaningful differences in blood pressure or renal function were observed. Despite this, patients with PTDM exhibited greater declines in GLS and EF, highlighting the impact of early metabolic disturbances on long‑term graft function.

Immunosuppressive therapy, particularly calcineurin inhibitors and corticosteroids, may contribute to both the development of PTDM and to direct myocardial effects, thereby amplifying metabolic stress on the graft.^15^ This dual impact complicates the interplay between metabolic and transplant‑related factors and may partially account for the myocardial dysfunction observed in patients with PTDM. Previous studies have demonstrated that even stable OHT recipients exhibit reduced myocardial deformation compared with healthy individuals, independent of overt coronary artery disease.^16^ In this context, our findings extend prior observations by identifying PTDM as an additional factor associated with impaired myocardial mechanics. Moreover, GLS has been shown to predict adverse cardiovascular events and mortality in OHT recipients, both with and without CAV,^17^ suggesting that the observed differences may have prognostic relevance.

In exploratory sex‑specific analyses, women consistently demonstrated more favorable GLS values than men, consistent with previous reports.^18^ The PTDM-associated impairment in GLS appeared more pronounced among women, with significantly lower GLS at both 3- and 5-year follow-up. This pattern is consistent with evidence that diabetes confers a disproportionately higher cardiovascular risk in women,^19^ and with experimental data demonstrating sex-specific molecular signatures of diabetic cardiomyopathy, including differences in mitochondrial function and myocardial fibrosis.^20^ Although our sex-specific analyses are exploratory and limited by sample size, they suggest that female heart transplant recipients with PTDM may represent a particularly vulnerable subgroup warranting further study.

We observed no differences in diastolic parameters between patients with and without PTDM, consistent with contemporary echocardiographic recommendations.^9^ Assessment of diastolic function in OHT patients remains challenging, as surgical alterations and post‑transplant physiology frequently affect conventional parameters.^21^, ^22^ Current recommendations emphasize the potential role of left atrial strain and LV strain rate during isovolumetric relaxation to improve diagnostic accuracy in this population.^22^, ^23^ Although these measures were not included in the present study, normal PAWP at 1-year argues against clinically significant diastolic dysfunction at early follow‑up. Nonetheless, diastolic abnormalities may evolve later in the post‑transplant course, particularly among patients with PTDM.

### Clinical implications

Several potentially modifiable factors may influence this trajectory and warrant prospective evaluation. Optimization of immunosuppressive therapy, including steroid-minimization strategies and individualized tacrolimus exposure, has been associated with a lower incidence of PTDM and may mitigate downstream myocardial effects.^3^ In parallel, SGLT-2 inhibitors have transformed the management of diabetes and heart failure in non-transplant populations and are increasingly being evaluated in solid organ transplant recipients, with emerging data suggesting acceptable safety and metabolic benefit after heart transplantation.^24^, ^25^ Notably, none of the patients in our cohort received SGLT-2 inhibitors during follow-up, reflecting both the era of recruitment and historical caution toward novel glucose-lowering therapies in transplant recipients. Whether these agents, or other cardiometabolic therapies such as glucagon-like peptide-1 receptor agonists, can preserve graft function in patients with PTDM represents a logical next step in transplant cardiology.

### Limitations

This study has several limitations. First, it is conducted at a single center with a relatively modest sample size, which may limit generalizability. Second, immunosuppressive regimens, including corticosteroid therapy, change over time and may confound metabolic outcomes. Third, right heart catheterization was performed only at 1-year, limiting longitudinal hemodynamic assessment. Data on rejection history and donor-specific antibodies were not consistently available and were not included, representing a potential source of unmeasured confounding. Additionally, sex‑specific analyses are limited by the small number of patients and should therefore be interpreted with caution. The modest cohort size underscores the need for larger, multicenter investigations to validate these findings. One such study, METAB HTX,^26^ may provide further insight into these mechanisms.

## Conclusion

Post-transplant diabetes diagnosed at 1-year after heart transplantation is associated with a distinct pattern of myocardial dysfunction, characterized by early and persistent impairment in global longitudinal strain followed by a progressive decline in ejection fraction over 5 years. Because this trajectory appears independent of CAV, our findings support the concept of a metabolically allograft dysfunction that is, in principle, modifiable. The absence of SGLT-2-inhibitor use in our cohort highlights an opportunity to evaluate whether contemporary cardiometabolic therapies may preserve graft function in this high-risk population.

## Financial disclosures

O.B. has received speaker and/or consulting fees from: AstraZeneca, Boehringer Ingelheim, Bayer, Orion Pharma, Bristol Myers Squibb, Novartis, Pfizer, Abbott Laboratories, and Pharmacosmos.

## Funding

O.B. is funded by SUS Fonder, Hjelms Stiftelse, and Hains Stiftelse. A.W.E, A.I., and O.B. are funded by USVE funding, Region Skåne. A.L. is funded by the Swedish governmental funding of clinical research (ALF). A.I. has received unrestricted grants from the Swedish Association for Cardiac Nurses and Allied Professionals.

## Declaration of Generative AI and AI-assisted technologies in the writing process

During the preparation of this work, the authors used Grammarly and Microsoft tools to improve grammar and linguistic clarity. These tools were not used for data analysis, interpretation, or scientific reasoning. All scientific content was developed by the authors, who take full responsibility for the manuscript.

## Declaration of competing interest

The authors declare the following financial interests/personal relationships which may be considered as potential competing interests: O.B. has received speaker and/or consulting fees from: AstraZeneca, Boehringer Ingelheim, Bayer, Orion Pharma, Bristol Myers Squibb, Novartis, Pfizer, Abbott Laboratories, and Pharmacosmos.

## References

[1] Kim I.C., Youn J.C., Kobashigawa J.A. (2018). The past, present and future of heart transplantation. Korean Circ J.

[2] Kim H.J., Jung S.H., Kim J.J. (2017). New-onset diabetes mellitus after heart transplantation- incidence, risk factors and impact on clinical outcome. Circ J Offl J Jpn Circ Soc.

[3] Newman J.D., Schlendorf K.H., Cox Z.L. (2022). Post-transplant diabetes mellitus following heart transplantation. J Heart Lung Transplant.

[4] Shindler D.M., Kostis J.B., Yusuf S., Quinones M.A., Pitt B., Stewart D. (1996). Diabetes mellitus, a predictor of morbidity and mortality in the Studies of Left Ventricular Dysfunction (SOLVD) Trials and Registry. Am J Cardiol.

[5] Devereux R.B., Roman M.J., Paranicas M. (2000). Impact of diabetes on cardiac structure and function: the strong heart study. Circulation.

[6] Ingvarsson A., Werther Evaldsson A., Waktare J. (2018). Normal reference ranges for transthoracic echocardiography following heart transplantation. J Am Soc Echocardiogr Offl Publ Am Soc Echocardiogr.

[7] Association AD. 2. Classification and Diagnosis of Diabetes: Standards of Medical Care in Diabetes—2021 44 Diabetes Care2020.

[8] Lang R.M., Badano L.P., Mor-Avi V. (2015). Recommendations for cardiac chamber quantification by echocardiography in adults: an update from the American Society of Echocardiography and the European Association of Cardiovascular Imaging. J Am Soc Echocardiogr Offl Publ Am Soc Echocardiogr.

[9] Nagueh S.F., Smiseth O.A., Appleton C.P. (2016). Recommendations for the evaluation of left ventricular diastolic function by echocardiography: an update from the American Society of Echocardiography and the European Association of Cardiovascular Imaging. Eur Heart J Cardiovasc Imaging.

[10] Humbert M., Kovacs G., Hoeper M.M. (2022). ESC/ERS Guidelines for the diagnosis and treatment of pulmonary hypertension: developed by the task force for the diagnosis and treatment of pulmonary hypertension of the European Society of Cardiology (ESC) and the European Respiratory Society (ERS). Endorsed by the International Society for Heart and Lung Transplantation (ISHLT) and the European Reference Network on rare respiratory diseases (ERN-LUNG). Eur Heart J.

[11] Vest A.R., Cherikh W.S., Noreen S.M., Stehlik J., Khush K.K. (2022). New-onset diabetes mellitus after adult heart transplantation and the risk of renal dysfunction or mortality. Transplantation.

[12] Feng K.Y., Henricksen E.J., Wayda B. (2021). Impact of diabetes mellitus on clinical outcomes after heart transplantation. Clin Transplant.

[13] Alizadehasl A., Mokhayeri M., Sohani Z., Zamanian M.Y., Shahbazi P., Borzouei S. (2025). A comprehensive review of two-dimensional speckle-tracking echocardiography in assessing right and left ventricular function in diabetic patients. Clin Cardiol.

[14] Jia G., Hill M.A., Sowers J.R. (2018). Diabetic cardiomyopathy: an update of mechanisms contributing to this clinical entity. Circ Res.

[15] Attachaipanich T., Chattipakorn S.C., Chattipakorn N. (2024). Cardiovascular toxicities by calcineurin inhibitors: cellular mechanisms behind clinical manifestations. Acta Physiol.

[16] Syeda B., Höfer P., Pichler P. (2011). Two-dimensional speckle-tracking strain echocardiography in long-term heart transplant patients: a study comparing deformation parameters and ejection fraction derived from echocardiography and multislice computed tomography. Eur J Echocardiogr J Working Group Echocardiogr Eur Soc Cardiol.

[17] Clemmensen T.S., Løgstrup B.B., Eiskjær H., Poulsen S.H. (2015). Evaluation of longitudinal myocardial deformation by 2-dimensional speckle-tracking echocardiography in heart transplant recipients: relation to coronary allograft vasculopathy. J Heart Lung Transplant.

[18] Ingvarsson A., Werther-Evaldsson A., Smith G.J. (2019). Impact of gender on echocardiographic characteristics in heart transplant recipients. Clin Physiol Funct Imaging.

[19] Peters S.A., Huxley R.R., Woodward M. (2014). Diabetes as risk factor for incident coronary heart disease in women compared with men: a systematic review and meta-analysis of 64 cohorts including 858,507 individuals and 28,203 coronary events. Diabetologia.

[20] Toedebusch R., Belenchia A., Pulakat L. (2018). Diabetic cardiomyopathy: impact of biological sex on disease development and molecular signatures. Front Physiol.

[21] Steding-Ehrenborg K., Nelsson A., Hedström E. (2024). Diastolic filling in patients after heart transplantation is impaired due to an altered geometrical relationship between the left atrium and ventricle. J Am Heart Assoc.

[22] Robinson S., Ring L., Oxborough D. (2024). The assessment of left ventricular diastolic function: guidance and recommendations from the British Society of Echocardiography. Echo Res Pract.

[23] Nagueh S.F., Sanborn D.Y., Oh J.K. (2025). Recommendations for the evaluation of left ventricular diastolic function by echocardiography and for heart failure with preserved ejection fraction diagnosis: an update from the American Society of Echocardiography. J Am Soc Echocardiogr.

[24] Raven L.M., Greenfield J.R., Jabbour A., Macdonald P.S., Muir C.A. (2025). Sodium glucose cotransporter 2 inhibitors are associated with renal stabilization in heart transplantation. JHLT Open.

[25] Sammour Y., Nassif M., Magwire M. (2021). Effects of GLP-1 receptor agonists and SGLT-2 inhibitors in heart transplant patients with type 2 diabetes: initial report from a cardiometabolic center of excellence. J Heart Lung Transplant.

[26] Polzin A., Scheiber D., Voss F. (2025). METAB-HTX: prospective, longitudinal cohort study evaluating cardiac and systemic metabolism after heart transplantation. ESC Heart Fail.

